# The Proposed Vitamin D–Homocysteine Relationship in Cardiometabolic Health: A Narrative Review of Mechanisms, Evidence, and Research Phenotypes

**DOI:** 10.3390/nu18193167

**Published:** 2026-09-25

**Authors:** Yun-Ah Lee, Sung-Goo Kang

**Affiliations:** Department of Family Medicine, St. Vincent’s Hospital, College of Medicine, The Catholic University of Korea, Seoul 16247, Republic of Korea; cool0216@catholic.ac.kr

**Keywords:** vitamin D, homocysteine, 25-hydroxyvitamin D, one-carbon metabolism, cardiovascular disease, Mendelian randomization, endothelial dysfunction, precision nutrition, hypothesis-generating biomarker profiles

## Abstract

The clinical relevance of a proposed relationship between vitamin D status and homocysteine remains uncertain. This narrative review integrates mechanistic, observational, Mendelian randomization (MR), and trial evidence. A documented supplementary search of PubMed and Europe PMC was executed on 21 September 2026 for publications through 31 July 2026; no formal risk-of-bias assessment or quantitative synthesis was performed. Experimental studies support vitamin D-dependent regulation of homocysteine metabolism and homocysteine-related suppression of vitamin D signaling, but reciprocal regulation has not been established in humans. Human biomarker associations and supplementation effects on homocysteine are heterogeneous; two frequently cited intervention publications report the same trial. Additional longitudinal and multibiomarker studies provide preliminary outcome associations, without externally validating the proposed four-category classification or its treatment utility. Homocysteine MR signals are more consistent for stroke than coronary disease, subject to validity and multiplicity concerns. Vitamin D MR is predominantly null, including the republished stratified analysis, and large supplementation trials have not demonstrated consistent cardiovascular benefit. A combined folate–vitamin D cognitive trial did not demonstrate its primary intention-to-treat benefit and does not validate vascular treatment selection. Clinical outcome trials carry greater weight for treatment decisions than molecular plausibility or biomarker co-occurrence. The proposed classification and explanatory models remain hypothesis-generating: a clinically meaningful reciprocal axis and differential benefit from phenotype-guided combined nutritional intervention are unproven.

## 1. Introduction

Vitamin D has traditionally been understood as a key regulator of calcium–phosphate homeostasis and skeletal health. Over recent decades, interest has expanded toward its extra-skeletal roles, including immune regulation, inflammation, insulin resistance, adipose tissue biology, endothelial function, vascular calcification, and cardiovascular disease [1,2,3,4]. This broader interest has been driven by observational studies showing that lower circulating 25-hydroxyvitamin D [25(OH)D] concentrations are frequently associated with cardiometabolic risk factors and adverse cardiovascular outcomes [4,5]. Nevertheless, interpretation remains controversial because large randomized controlled trials and trial-level syntheses have not consistently demonstrated that vitamin D supplementation reduces major cardiovascular events [6,7,8,9,10,11].

Homocysteine is another biomarker with a long and complex cardiovascular history. As a sulfur-containing amino acid generated during methionine metabolism, homocysteine occupies a central position at the intersection of remethylation and transsulfuration pathways, and its metabolism depends substantially on folate, vitamin B12, vitamin B6, renal function, and genetic determinants of one-carbon metabolism [12,13]. Elevated homocysteine has been linked to endothelial dysfunction, oxidative stress, thrombosis, vascular stiffness, cognitive decline, chronic kidney disease, and cardiovascular risk [14,15,16]. Yet, as with vitamin D, intervention trials have complicated the interpretation of observational associations: folic acid and B vitamins reliably lower homocysteine, but biochemical improvement has not consistently translated into reductions in coronary heart disease, mortality, or major vascular events [17,18,19,20,21,22].

The relationship between vitamin D and homocysteine therefore sits at the intersection of two controversial fields. Observational studies [23,24,25,26,27,28] and linked biomarker-focused trial reports [29,30] suggest a relationship between 25(OH)D and homocysteine, including a recent study in asymptomatic Korean adults in which 25(OH)D was negatively associated with serum homocysteine after adjustment for covariates [23]. This association should not be read as a simple causal sequence in which low vitamin D directly raises homocysteine, which in turn causes cardiovascular disease, such that supplementation prevents events. Such a linear narrative is vulnerable to the twin observations that vitamin D supplementation and homocysteine lowering have each failed to reduce hard cardiovascular outcomes in unselected populations [6,7,8,9,10,11,12,13,14,15,16,17,18,19,20,21,22].

Existing systematic reviews and trial syntheses already address whether vitamin D supplementation or homocysteine-lowering therapy reduces cardiovascular events [6,7,8,9,10,11,17,18,19,20,21,22]. The narrower question addressed here is whether evidence concerning the relationship between the two biomarkers supports interpreting them together. This requires separating reciprocal molecular effects, human biomarker associations, the causal-inference evidence for each exposure, and treatment effects on clinical outcomes. This review primarily synthesizes mechanistic hypotheses and their human evidentiary limits; risk stratification is a secondary research question. When retained, “proposed axis” denotes a testable organizing hypothesis. Establishing an axis would require reproducible reciprocal effects in humans, temporality and mediation across relevant tissues, and evidence of clinical relevance beyond shared determinants. These requirements have not been met. A narrative synthesis can examine the compatibility and limitations of these evidence streams, but cannot determine whether a neutral trial reflects insufficient duration or power, inadequate targeting, or absence of benefit.

This review therefore asks which vitamin D–homocysteine links are directly supported, where findings disagree, and what remains to be tested. Its intended contribution is a critical evidence map linking the mechanistic literature to the limitations of combined-biomarker interpretation, rather than a new pooled effect estimate or a resolution of the neutral-trial findings. The proposed phenotypes and explanatory models organize research questions; they are not conclusions established by the synthesis.

## 2. Methods: Literature Search and Narrative Synthesis Approach

This narrative review combines an initial reference set with a documented supplementary search undertaken during revision. It is not a systematic review or meta-analysis, and no protocol was registered. The supplementary search was executed on 21 September 2026 in PubMed (NCBI E-utilities) and Europe PMC (REST API), restricting publication dates to 1800 through 31 July 2026. The literature cutoff is distinct from the execution date. The earlier narrative search cannot be retrospectively reconstructed from the available search documentation; the counts and reproducible strategies reported here refer specifically to the supplementary search.

The broad PubMed strategy combined vitamin D terms, including relevant MeSH headings, metabolites and VDR, with homocysteine/hyperhomocysteinemia terms. Three nested subsets added vascular terms, causal/intervention/nutritional terms, or joint-profile terms. Europe PMC provided an overlapping title/abstract search for “vitamin D” and “homocysteine”; it was not treated as an independent literature universe. No language, age, or design filters were applied during retrieval. The search scope and execution are described in Supplementary Material S1. Complete search strategies, deduplication and selection procedures, source availability and qualitative appraisal are provided in Supplementary Materials S2–S4, respectively. Scopus, Web of Science, and Google Scholar were not rerun in this supplementary audit.

The broad searches returned 487 PubMed and 371 Europe PMC records. Deduplication by PMID, normalized DOI, and then normalized title plus year removed 344 duplicate database records, leaving 514 unique records. Nested PubMed subsets returned 160 vascular, 273 causal/intervention/nutritional, and 55 joint-profile records; these are subsets of the 487 and were not added to the total. Separate publications from the same study were retained as publications and linked at study level rather than counted as independent trials.

Selection prioritized primary evidence directly assessing reciprocal molecular pathways, paired human biomarkers, joint vascular associations, or vitamin D-containing interventions measuring homocysteine. Adult cardiometabolic relevance, design, temporality, covariate control, and precision guided selection, not statistical significance or effect direction. AI tools were used to assist initial title/abstract screening, with original-source inspection for detailed additions. Both authors (Y.-A.L. and S.-G.K.) reviewed all preliminary screening decisions for the 514 unique records, including selection for detailed synthesis, retention as contextual evidence, and non-selection. The corresponding author (S.-G.K.) additionally verified the publications selected for inclusion in the detailed synthesis. Final inclusion decisions were made by the authors. Of 514 records, 386 were not selected because of topic, population, language, or report type; 105 related records were retained in the contextual audit without a new detailed synthesis entry; and 23 publications received detailed treatment, comprising 12 already tabulated and 11 added during revision. These are representative narrative selections, not an exhaustive set of eligible studies.

The paired-term audit specifically checked coverage of the proposed relationship. Landmark single-biomarker outcome trials, MR studies, and reviews were retained as purposefully selected contextual evidence and were not exhaustively retrieved by this search. Full text was examined for eight of the eleven added reports; three additions were limited to verified primary abstracts, as identified in Supplementary Table S1. Table 1, Table A1, Table A2 and Table A3 and Supplementary Table S3 provide study-specific qualitative appraisal of directness, design, confounding, temporality, randomization/reporting limitations, precision, and consistency. No formal risk-of-bias instrument, GRADE assessment, or pooling was applied. Selective narrative coverage, a focused search, and incomplete full-text access remain limitations; the supplementary audit improves transparency without establishing systematic-review completeness.

For questions of clinical benefit, randomized trials with patient-important outcomes and their syntheses were given the greatest interpretive weight, within the populations and interventions studied. MR provided complementary causal-inference evidence subject to instrument validity, pleiotropy, and population assumptions. Observational studies informed biomarker co-occurrence and candidate prognostic relationships; cell and animal studies informed biological plausibility. Biomarker changes were not treated as evidence of clinical benefit, and models proposed by the authors were distinguished from empirical findings. These are interpretive principles, not a formal certainty-of-evidence grading system.

### Use of Generative Artificial Intelligence in Manuscript Preparation

The authors used ChatGPT with the GPT-6 Astra model (OpenAI, San Francisco, CA, USA) and Claude (Anthropic, San Francisco, CA, USA) as supporting tools for language editing, manuscript organization and drafting, and conceptual figure design. During revision, ChatGPT also assisted with scripted supplementary searches, deduplication, preliminary title/abstract screening, original-source checking, study-level extraction, cross-domain evidence comparison, and refinement of the proposed explanatory and research frameworks. The authors retained responsibility for study selection, verification of extracted information, scientific interpretation, and approval of the final manuscript; their review of screening decisions is described in Section 2. AI outputs were not treated as evidence or as a substitute for assessment of original sources. No new participant-level statistical analyses were performed.

## 3. Biological Foundations and Shared Determinants

### 3.1. Vitamin D Metabolism and Signaling

Vitamin D status is usually assessed by circulating 25(OH)D. Vitamin D is obtained through cutaneous synthesis after ultraviolet-B exposure and through dietary intake or supplementation, is hydroxylated in the liver to 25(OH)D, and is then converted by 1-alpha-hydroxylase (CYP27B1) to 1,25-dihydroxyvitamin D [1,25(OH)2D], the active hormonal form. The active metabolite binds the vitamin D receptor (VDR), a nuclear receptor regulating genes involved in calcium metabolism, immune function, cellular differentiation, and inflammatory signaling [1,2,3]. CYP24A1 catabolizes both 25(OH)D and 1,25(OH)2D and is central to vitamin D homeostasis [2,3]; its regulation is important to the mechanistic discussion below.

The biological interpretation of 25(OH)D is complex. Circulating concentrations reflect sunlight, diet, supplement use, adiposity, season, skin pigmentation, renal and hepatic function, inflammation, and genetics, and the same 25(OH)D concentration may not imply equivalent tissue-level activity across individuals, because local conversion, VDR expression, vitamin D binding protein, and downstream responsiveness differ across tissues and disease states [3,4,31,32,33,34,35,36]. Accordingly, 25(OH)D is a marker of circulating vitamin D status, not a validated surrogate for tissue-specific VDR expression or transcriptional activity. Experiments using active vitamin D or manipulating VDR cannot be equated with an observational difference in serum 25(OH)D.

### 3.2. Homocysteine Metabolism

Homocysteine is generated from methionine and can be remethylated to methionine or committed via transsulfuration. Remethylation requires 5-methyltetrahydrofolate as methyl donor and vitamin B12 as cofactor for methionine synthase, with betaine-homocysteine methyltransferase providing a hepatic-renal alternative; transsulfuration toward cystathionine and cysteine depends on vitamin B6. Serum homocysteine is therefore influenced by methionine intake, folate, vitamin B12, vitamin B6, choline/betaine metabolism, renal function, thyroid function, medications, smoking, alcohol, and genetic variants such as MTHFR C677T [12,13,35,37].

This context is important when interpreting the axis. Hyperhomocysteinemia cannot be attributed to vitamin D deficiency alone; in many individuals it primarily reflects B-vitamin insufficiency, impaired renal clearance, genetic variation, smoking, hypothyroidism, or chronic inflammation [12,13,37,38]. Conversely, low 25(OH)D may reflect limited sun exposure, obesity-related dilution, dietary insufficiency, malabsorption, liver disease, or systemic illness [31,32,33,34]. Clinically meaningful interpretation therefore requires attention to these shared determinants rather than a single-biomarker explanation.

### 3.3. Shared Biological Context

Vitamin D deficiency and hyperhomocysteinemia frequently coexist in states of cardiometabolic stress. Aging, obesity, diabetes, chronic kidney disease, physical inactivity, smoking, poor diet, and inflammation influence both biomarkers. In obesity, lower 25(OH)D has been attributed to volumetric dilution, sequestration in adipose tissue, reduced outdoor activity, and altered metabolism [31,32,33,34], while abdominal obesity, insulin resistance, and multimorbidity have been associated with altered one-carbon metabolism and higher homocysteine [26,39,40,41,42]. Two explanations must therefore be distinguished from the outset: direct regulation between the pathways, and shared determinants that generate biomarker co-occurrence without reciprocal regulation. The established effects of renal, nutritional, and adiposity-related factors make the latter a substantial alternative, not merely a residual caveat. Whether the combined profile adds prognostic information beyond these shared determinants is an empirical question; neutral supplementation trials do not establish that combined selection would improve outcomes [6,7,8,9,10,11,12,13,14,15,16,17,18,19,20,21,22].

## 4. Preclinical Evidence for Proposed Bidirectional Crosstalk

Experimental studies provide a rationale for investigating reciprocal regulation. The term “axis” is used here as an organizing hypothesis; the studies below do not establish a self-sustaining feedback loop or its clinical importance in humans.

Table A1 maps the three principal mechanistic reports [43,44,45] by species, tissue or cell type, perturbation, pathway, direction, outcome, and limitations. Their myocardial findings do not establish the same regulatory sequence in human endothelium, liver, kidney, or cerebrovascular tissue. Renal CYP24A1 changes in the rat study [45] provide a specific tissue observation, not validation of a systemic human feedback circuit.

### 4.1. Vitamin D Signaling as a Regulator of Homocysteine Metabolism

A key question is whether vitamin D can regulate homocysteine metabolism. Experimental evidence indicates that vitamin D/VDR signaling upregulates methionine synthase in an Nrf2-dependent manner, thereby reducing myocardial homocysteine in models of hyperhomocysteinemia [43], with related mouse and cardiomyocyte work implicating NFE2L2-mediated MTHFR regulation in homocysteine-induced myocardial inflammation [44]. Because methionine synthase catalyzes remethylation of homocysteine to methionine, these findings place vitamin D signaling upstream of a remethylation pathway in the experimental systems studied. On its own this does not establish that supplementation meaningfully lowers homocysteine in humans or reduces events, but it defines a plausible molecular route by which vitamin D signaling could affect homocysteine metabolism [43,44]; the corresponding human trial evidence, which is biomarker-level rather than outcome-level, is examined in Section 7.3.

The forward hypothesis also has earlier support outside myocardium. Kriebitzsch et al. showed VDR-dependent regulation of cystathionine β-synthase (CBS) and transsulfuration in murine osteoblast models and a human osteosarcoma-derived cell line, together with an observational analysis in older adults [46]. This broadens the experimental context but does not establish reciprocal regulation in human vascular tissue or a clinically meaningful circulating axis (Supplementary Table S1).

### 4.2. Homocysteine as a Suppressor of Vitamin D Signaling

Evidence for the reverse direction comes from methionine-fed rats and homocysteine-exposed cardiomyocytes. These experiments indicate that homocysteine can reduce 1,25(OH)2D levels and repress myocardial VDR expression through CYP24A1 upregulation and miR-125b-5p-related regulation, contributing to cardiac hypertrophy [45]. Combining these observations with the forward pathway generates a feedback hypothesis, but reciprocal effects reported in separate experiments do not demonstrate an integrated feedback loop in humans. Because the evidence is still predominantly preclinical, the cautious interpretation is that emerging mechanistic work supports bidirectional crosstalk, not that a clinically established bidirectional causal axis has already been proven [43,44,45].

### 4.3. Oxidative Stress, Inflammation, and Endothelial Dysfunction as Convergent Pathways

Beyond the proposed reciprocal regulation, studies of each exposure implicate overlapping vascular injury pathways. Homocysteine promotes reactive oxygen species generation, impaired nitric oxide bioavailability, endothelial injury, prothrombotic states, and vascular inflammation [14,47,48], whereas vitamin D signaling has been studied in relation to anti-inflammatory and antioxidant pathways and to modulation of the renin–angiotensin system [2,3,4,43,44]. These mechanisms are not specific to the proposed axis. Their overlap provides candidate intermediates for study, but does not establish additive or synergistic vascular injury from the combined phenotype, or a stronger effect in inflammatory or renal-metabolic subgroups [31,32,33,34,39,40,41,42,48].

### 4.4. Why Bidirectionality Matters

The strongest mechanistic evidence concerns molecular responses under experimental perturbation. Its principal limitation is the translation from myocardial cells and animal models to circulating biomarker concentrations and clinical outcomes in heterogeneous human populations. Limited human intervention reports provide preliminary evidence of a forward biomarker response (Section 7.3), but do not test the reciprocal pathway or establish that the experimental mechanism mediates that response. Figure 1 retains the proposed reciprocal mechanisms and shared vascular pathways, while identifying their evidence sources. Dashed connections denote the authors’ unvalidated integration; the clinical associations are not linked by a causal arrow to the mechanistic pathways. Low circulating 25(OH)D should not be equated with the experimental perturbations of vitamin D/VDR signaling.

## 5. Observational Evidence Linking Vitamin D Status and Homocysteine

### 5.1. General Adult and Health-Screening Populations

The studies in Table A2 differ substantially in design and covariate coverage [23,24,25,26,27,28,38]. In 14,630 NHANES adults, Amer and Qayyum reported an inverse association below 25(OH)D of 21 ng/mL after adjustment including folate, vitamin B12, renal function, and CRP; above that concentration the association was null [24]. In 1375 Korean screening participants, the association persisted after creatinine and hs-CRP adjustment (β = −0.033, *p* < 0.001), but folate, vitamin B12, and season were not included [23]. A separate Korean study adjusted for age, sex, BMI, hypertension, and diabetes, providing less complete control of the major biochemical determinants [27]. These findings support co-occurrence but are not equivalent demonstrations of an independent relationship. Cross-sectional data cannot establish directionality or added predictive value.

### 5.2. Older Adults and Vascular-Neurocognitive Aging

Older adults are especially relevant because aging reduces cutaneous vitamin D synthesis and outdoor activity and is accompanied by nutritional insufficiency, declining renal function, polypharmacy, frailty, sarcopenia, cognitive vulnerability, and vascular aging—factors affecting both biomarkers [12,13,37,38]. Concurrent low vitamin D and high homocysteine in older adults may therefore reflect shared determinants rather than a single deficiency. Recent work exploring combined vitamin D deficiency and hyperhomocysteinemia as a combined biomarker category in older hypertensive patients, particularly in relation to executive function [38], provides a measurable combined-biomarker construct, but an association with executive function in one population does not establish prediction of incident stroke or cardiovascular events.

### 5.3. Obesity, Diabetes, and Chronic Kidney Disease

Cardiometabolic populations illustrate why biomarker co-occurrence may reflect shared determinants rather than a direct causal link. In obesity, lower 25(OH)D arises partly from volumetric dilution, sequestration, and altered metabolism, while chronic low-grade inflammation, insulin resistance, and adipokine dysregulation may influence homocysteine [31,32,33,34,39,40,41,42,48]. In diabetes, oxidative stress, endothelial dysfunction, renal microvascular injury, medications, and altered nutrient metabolism affect both markers, so interpreting combined low vitamin D/high homocysteine as evidence of greater oxidative or endothelial burden requires direct validation and remains susceptible to confounding [4,26,39,40,41,42,48]. Chronic kidney disease is perhaps the most important context: the kidney participates in vitamin D activation (CYP27B1) and homocysteine clearance, so low 25(OH)D and elevated homocysteine commonly coexist as renal function declines, but this coexistence may be driven primarily by renal dysfunction [1,2,3,12]. Renal function is therefore a major shared determinant and a potential confounder, depending on the causal question and timing of measurement (Section 10). Whether it also modifies a vitamin D–homocysteine association or treatment effect requires prespecified interaction testing rather than assumption.

### 5.4. Interpretive Summary

An inverse association recurs, but there is no uniform effect size across healthy screening populations, older patients with established disease, and angiography cohorts. Among the original tabulated biomarker studies, the NHANES analysis provides the clearest persistence after simultaneous renal, folate, vitamin B12, and inflammatory adjustment, although season was not part of the reported model [24]. The preventive-program analysis adjusted for B12 and showed similar findings after restriction to preserved renal function, but did not establish independence from folate, season, or concurrent behavioral changes [25]. The large Chinese clinical series mainly used correlations and stratification rather than comprehensive multivariable control [26]. In the angiography study, subgroup associations with disease severity were not accompanied by significant vitamin D–homocysteine interaction tests [28]. Table A2 makes these differences explicit. None of these analyses alone establishes a generalizable joint vascular effect or validates routine paired testing.

The supplementary search identified both supporting and null population evidence. In 1928 Canadian survey participants, 25(OH)D was not associated with homocysteine after adjustment including season and adiposity [56]. In the Multi-Ethnic Study of Atherosclerosis, a 10 ng/mL lower 25(OH)D was associated with 3.7% higher homocysteine (95% CI 3.0–4.3) among 6443 participants, with renal and other covariate adjustment and sensitivity analyses for estimated folate/B12 intake [57]. Dietary intake adjustment is not equivalent to measured B-vitamin status. These differences reinforce heterogeneous association rather than a uniform biomarker effect.

## 6. Causal Inference: Mendelian Randomization

Because observational associations are vulnerable to residual confounding and reverse causation, Mendelian randomization (MR), which uses germline genetic variants as instrumental variables for lifelong exposure, offers a complementary approach to causal inference. The available MR evidence suggests that the causal roles of homocysteine and vitamin D are neither uniform across cardiovascular outcomes nor symmetrical between the two markers. These analyses address the individual exposures; they do not directly test reciprocal regulation or the incremental value of a joint phenotype.

### 6.1. Homocysteine: An Outcome-Specific MR Signal

MR studies provide more consistent, although not uniformly conclusive, evidence for a causal contribution of higher homocysteine to stroke, particularly small-vessel disease, rather than a uniform effect across cardiovascular outcomes. In an MR analysis using genetic consortia, the UK Biobank, and FinnGen, each standard-deviation increase in genetically predicted total homocysteine had an odds ratio of 1.11 (95% CI 1.03–1.21; *p* = 0.008) for any stroke, but this did not meet the study’s multiplicity-corrected threshold of *p* < 0.001; coronary artery disease and atrial fibrillation results were null [49]. A 2025 systematic review of 33 MR studies identified recurring signals for ischemic and small-vessel stroke while emphasizing heterogeneity and uncertainty for most non-cerebrovascular outcomes [50]. An outcome-wide umbrella review similarly found stroke and small-vessel occlusion stroke among the outcomes with comparatively robust and adequately powered causal evidence [51]. In a 2025 multiancestry analysis, genetically proxied lower MTHFR activity was associated with small-vessel stroke in East Asian and European populations, but not with large-artery or cardioembolic stroke, further localizing the signal rather than establishing a pan-cardiovascular effect [52]. Genetic and clinical evidence linking homocysteine to hypertension also supports attention to the cerebrovascular–hypertensive domain [39]. Overall, the evidence is compatible with an outcome-specific causal contribution, but its magnitude and generalizability remain uncertain.

### 6.2. Vitamin D: Predominantly Null MR Evidence

MR evidence for vitamin D is predominantly null for cardiovascular outcomes, but it is not entirely uniform. A large study of approximately 99,012 Chinese and 106,911 European adults found no consistent association between genetically higher 25(OH)D and major vascular outcomes, apart from isolated population-specific findings [53]. The republished dose–response analysis found inverse observational associations at low 25(OH)D, but its population-wide and stratified genetic analyses were null for coronary heart disease, stroke, and all-cause mortality across observed vitamin D levels [54]. It therefore does not support a protective genetic effect confined to low vitamin D status. The 2024 report replaced a retracted 2021 analysis after concerns about the original non-linear MR method; the interpretation here follows the republished results. A separate 2025 observational-mediation and MR study reported an inverse association between genetically predicted 25(OH)D and stroke (IVW odds ratio 0.92, 95% CI 0.85–0.99) [55]. This selected positive finding warrants replication but does not outweigh the broader null pattern or establish supplementation benefit.

### 6.3. The Asymmetry and Its Interpretive Value

Homocysteine has a more consistent MR signal for stroke and small-vessel disease than for coronary outcomes, whereas vitamin D findings are predominantly null [49,50,51,52,53,54,55]. This difference argues against treating the two biomarkers as equally supported causal components of a vascular pathway. MR estimates remain vulnerable to invalid instruments, horizontal pleiotropy, and differences in ancestry or exposure modeling; genetically proxied lifelong exposure is also not equivalent to supplementation begun in adulthood. A diabetes-specific MR analysis suggested a homocysteine–coronary association [58], but comparison with general-population studies is not itself proof of effect modification. Overall, MR informs exposure-specific hypotheses, not the existence of the combined axis or the superiority of phenotype-guided treatment.

Validity and precision require study-specific consideration. Yuan et al. used 14 homocysteine variants explaining 6% of exposure variance, with weighted-median, MR-Egger, MR-PRESSO, and exclusion analyses for potentially pleiotropic variants; the stroke results were suggestive after accounting for multiple testing [49]. Huang et al. reported instrument F statistics above 15, examined synthesis-related variants separately from transport-related variants, and found broadly null vascular effects in Chinese and European adults [53]. These checks reduce particular concerns but do not establish the exclusion restriction or eliminate ancestry-related differences.

The republished analysis [54] used biologically selected vitamin D loci, ancestry adjustment, and doubly-ranked stratification. Its null findings across strata differed from the original residual-stratification results, illustrating sensitivity to the assumption that genetic effects on the exposure are constant. Direct stratification on measured 25(OH)D can introduce collider bias. The larger sample and event counts strengthen precision for average effects, while smaller strata still cannot exclude modest effects. By comparison, the selected positive IVW stroke estimate in Tsai et al. [55] was close to the null (*p* = 0.036); the observational arm is not an independent genetic replication. Differing exposure scales and stroke definitions preclude a direct comparison of the two odds ratios.

Across MR reports, instrument strength should be assessed with F statistics and explained variance, not sample size alone. Weak-instrument bias can differ with exposure–outcome sample overlap; reuse of large biobanks also limits independence between publications. Pleiotropy checks, ancestry matching and population-stratification control, overlap, endpoint-specific power, and multiplicity therefore constrain both positive and null conclusions. Absence of a significant pleiotropy test is not proof of valid instruments. Most analyses concern individual biomarkers; none of these results directly tests a joint vitamin D–homocysteine intervention or reciprocal human regulation.

## 7. Clinical Trial Evidence: Biomarker Effects and Cardiovascular Outcomes

### 7.1. Vitamin D Supplementation and Cardiovascular Outcomes

The major challenge to a therapeutic vitamin D narrative is the largely neutral cardiovascular trial evidence. In VITAL, vitamin D supplementation did not reduce major cardiovascular events versus placebo [6]. The Vitamin D Assessment Study and Finnish Vitamin D Trial also failed to demonstrate clear cardiovascular prevention in broad populations [8,9]. The D-Health trial suggested a possible reduction in major cardiovascular events, but the absolute difference was small and the confidence interval included a null finding [10]. Recent syntheses remain neutral: a 2026 meta-analysis of nine randomized trials involving 114,379 participants found no significant reduction in cardiovascular disease events (RR 0.95, 95% CI 0.88–1.04) or cardiovascular mortality [59], consistent with earlier reviews [7,11]. Many trials enrolled largely vitamin D–replete participants, which limits inference about severe deficiency, but this design feature does not establish that deficient subgroups would benefit. Routine supplementation of broad populations should therefore not be portrayed as an established cardiovascular strategy [6,7,8,9,10,11,59].

### 7.2. Homocysteine-Lowering Therapy and Cardiovascular Outcomes

A parallel pattern holds for homocysteine. Folic acid and B-vitamin supplementation reliably lowers circulating homocysteine, especially when baseline concentrations are elevated or folate status is inadequate [60], but clinical effects vary by outcome and population. HOPE-2 did not reduce its composite primary endpoint despite lowering homocysteine, although stroke was reduced [17]; NORVIT, SEARCH, and VITATOPS did not show consistent reductions in their major vascular endpoints [18,19,20]. A 2025 meta-analysis of 45 randomized trials reported reductions in stroke (RR 0.85, 95% CI 0.76–0.96) and a small reduction in overall cardiovascular disease, but not coronary heart disease or all-cause mortality; the wide prediction interval for stroke indicated substantial setting-to-setting uncertainty [22]. A 2026 meta-analysis in coronary heart disease likewise found lower homocysteine and reduced restenosis after combined B-vitamin supplementation, but no significant reduction in major cardiovascular events or mortality [61]. This evidence is broadly consistent with, but does not prove, an outcome-specific homocysteine effect. Baseline folate, achieved homocysteine reduction, renal function, cointerventions, and endpoint selection remain important candidate modifiers [22,61].

### 7.3. Vitamin D Supplementation and Homocysteine: Biomarker-Level Evidence in Humans

The human intervention literature is broader than the two frequently cited Al-Bayyari publications. Those reports [29,30] share the same registration, NCT03310307, and the later report explicitly describes a post hoc analysis involving the same participants; they are not independent replications (Table A3). A separate four-arm randomized study in 92 adults with type 2 diabetes reported Hcy reductions in vitamin D, chromium, and combined-treatment groups over four months [62]. Its verified abstract does not provide a precise between-group Hcy effect estimate or establish pathway mediation. These small biomarker trials do not demonstrate prevention of vascular events.

Null or less favorable biomarker findings must also be considered. A 2026 placebo-controlled trial randomized 40 adults with epilepsy to vitamin D3 2000 IU/day or placebo for 12 weeks; 34 completed follow-up. Homocysteine increased in both groups, with no significant difference in change (*p* = 0.290), in a setting that included cessation of B-vitamin supplements [63]. A CKD study comparing vitamin D dose regimens likewise did not report Hcy lowering [64]. Conversely, an H-type hypertension report described lower Hcy after vitamin D2 was added to folic acid and antihypertensive treatment, but its retrospective description, claimed random allocation, and inconsistent statistical reporting limit confidence [65]. Supplementary Table S1 details these reports. Thus, Hcy lowering is not a consistently reproduced consequence of vitamin D supplementation, and cointerventions and renal context require explicit consideration.

Combined nutritional treatment has also been studied outside vascular-event prevention. In 402 participants with mild cognitive impairment and vitamin D deficiency, folic acid plus vitamin D3 did not significantly improve the primary intention-to-treat cognitive outcome versus placebo or folic acid alone at 24 weeks [66]. This trial prevents a blanket claim that combined interventions have never been studied; it does not demonstrate preferential benefit in the proposed low-vitamin-D/high-Hcy phenotype or prevention of cardiovascular events.

### 7.4. A Context-Specific Exception: Baseline Folate Status and Stroke Prevention

A clinically important positive result should be considered alongside the neutral trials. In the China Stroke Primary Prevention Trial (CSPPT), conducted among hypertensive adults in a setting without mandatory folate fortification, adding folic acid to enalapril reduced first stroke relative to enalapril alone; the design also incorporated MTHFR genotype and baseline folate status [21]. The stroke endpoint and low-folate hypertensive setting are compatible with the outcome-specific MR pattern for homocysteine [49,50,52]. However, CSPPT tested folic acid added to antihypertensive therapy—not vitamin D correction, combined biomarker phenotyping, or dual supplementation. It therefore supports the possibility that baseline nutritional context and outcome selection influence benefit, but it does not validate the vitamin D–homocysteine axis as a treatment target or show that prior neutral trials failed because of targeting.

### 7.5. Why Biomarker Correction Does Not Always Reduce Events

Biomarker improvement and clinical benefit answer different questions. A biomarker may reflect disease or shared determinants rather than a modifiable cause, and changing its concentration need not alter vascular risk. Timing of treatment, baseline nutritional status, adherence, and concomitant therapies could also influence an intervention’s effect [6,7,8,9,10,11,12,13,14,15,16,17,18,19,20,21,22]. These are competing explanations to be evaluated with trial data, not established reasons to discount neutral outcomes. This review cannot determine whether inadequate duration or power accounts for a null result; the observed effect estimate and its confidence interval remain central. Intermediate vascular measures may inform early mechanistic research, but favorable changes cannot be assumed to predict fewer clinical events.

### 7.6. Implications for Phenotype-Based Trials

The clinical trial evidence does not support routine cardiovascular supplementation based on either biomarker in broad populations. A staged program could first validate combined profiles prospectively, then test a narrowly specified mechanism or biomarker response, and consider event-driven trials only after reproducible signals justify them. Measuring biological context is distinct from stratifying simultaneously by every covariate (Section 12). Until prospective validation and intervention evidence are available, the proposed profile should remain a research-stratification tool rather than a basis for routine dual testing or supplementation. Figure 2 organizes the clinical trial findings, exposure-specific MR and CSPPT context, and candidate phenotype-based designs into an author-proposed research framework. This sequence does not establish that inadequate targeting explains neutral trials or that phenotype-guided selection improves outcomes.

The evidence synthesis, including key limitations and interpretive implications, is presented in Table 1.

Across evidence types, the apparent divergence is partly a difference in the question being tested. Observational co-occurrence can arise from shared determinants and reverse causation; experimental perturbations show what can occur in a model; MR examines genetically proxied exposure; and trials test a specific intervention in a defined population. For cardiovascular treatment decisions, the largely neutral vitamin D outcome trials and predominantly null MR evidence deserve greater weight than inverse biomarker associations. The homocysteine–stroke findings and CSPPT support an exposure- and setting-specific interpretation, not the complete vitamin D–homocysteine model. Our synthesis therefore supports biological plausibility and biomarker co-occurrence, while leaving joint prognostic value, interaction, and treatment utility unresolved.

**Table 1:** Representative studies, quantitative findings, and qualitative evidentiary appraisal.

Evidence/Study	Design and Sample	Exposure/Comparison	Representative Finding	Confidence and Directness
Mechanistic [43,44,45]	Mouse/rat myocardium and cardiomyocytes; experiment sizes NR in verified abstracts	Vitamin D/VDR or Hcy perturbation	Nrf2–MTR/MTHFR and CYP24A1/miR-125b-5p–VDR effects; Table A1	Preclinical plausibility; human reciprocal regulation unvalidated.
Lee et al. [23]	Cross-sectional; 1375 Korean screening adults	Serum 25(OH)D and Hcy	Adjusted β −0.033; *p* < 0.001	Association only; B vitamins and season not adjusted. Table A2.
Yuan et al. [49]	Two-sample MR; Hcy discovery GWAS n = 44,147; outcome-specific consortium samples	14 Hcy variants; per 1-SD higher Hcy	Any stroke OR 1.11 (1.03–1.21), *p* = 0.008	Suggestive: did not meet multiplicity threshold *p* < 0.001; instrument assumptions remain.
Republished analysis [54]	MR; 386,406 European-ancestry participants; 18,166 stroke events	Per 10 nmol/L genetically higher 25(OH)D	Stroke OR 1.01 (0.97–1.05); CHD OR 0.98 (0.95–1.01); stratified results also null	Substantial evidence against a large average effect on these scales; small stratum-specific effects cannot be excluded.
VITAL [6]	Placebo-controlled RCT; n = 25,871; median 5.3 years	Vitamin D3 2000 IU/day	Major CVD HR 0.97 (0.85–1.12)	Direct outcome evidence: no demonstrated benefit in the studied population; not a joint-profile trial.
CSPPT [21]	Randomized trial; n = 20,702; median 4.5 years	Enalapril + folic acid vs. enalapril	First stroke 2.7% vs. 3.4%; HR 0.79 (0.68–0.93)	Direct, setting-specific folate benefit; indirect to the proposed combined relationship.
Al-Bayyari reports [29,30]	Small randomized trial and linked post hoc report; Table A3	Vitamin D3 vs. placebo	Hcy reduction; no vascular-event endpoint	Limited biomarker evidence; reports are not independent replication.
Tan et al. [38]	Cross-sectional; 452 older hypertensive inpatients	Both abnormalities vs. neither	Executive dysfunction OR 2.1 (1.5–2.9)	Exploratory, outcome-specific association; no prospective CVD prediction or treatment validation.

Abbreviations: 25(OH)D, 25-hydroxyvitamin D; Hcy, homocysteine; MR, Mendelian randomization; RCT, randomized controlled trial; OR, odds ratio; HR, hazard ratio. Effect scales and outcomes differ and are not pooled. Confidence descriptors are narrative judgments about the stated inference, not GRADE ratings. Table A1, Table A2 and Table A3 and Supplementary Table S1 provide detailed direct-link and adjacent evidence, including contrasting findings. NR means not reported in the accessible source used for extraction, not evidence that the original experiment had no sample-size information.

## 8. Proposed Hypothesis-Generating Classification of Combined Biomarker Profiles

### 8.1. Rationale for a Combined Phenotype Framework

A central proposal of this review is to study vitamin D and homocysteine not only as separate biomarkers but also as combined research phenotypes. Concordant abnormalities may reflect shared metabolic vulnerability, whereas discordant patterns may point toward distinct mechanisms. A simple 2 × 2 framework can be used for hypothesis generation (Table 2): normal/normal; low vitamin D/normal homocysteine; normal vitamin D/high homocysteine; and low vitamin D/high homocysteine. Direct prospective evidence for cardiovascular prediction is limited, intervention benefit has not been demonstrated, and the framework is not a validated clinical algorithm. Here “normal” means within a prespecified study reference range, not an established optimal vascular threshold. The four cells do not imply a validated ordering of cardiovascular risk.

The classification has preliminary empirical precedents rather than established clinical validity. A cross-sectional study in 452 older hypertensive inpatients reported executive-function associations and interaction [38], while the angiography study had null CAD interaction tests [28]. A nested case–control study with 1079 incident ischemic stroke cases and 1079 controls reported homocysteine as a modifier of the vitamin D association [67]. An NHANES analysis of 5459 hypertensive adults, including 2276 with H-type hypertension, related joint vitamin D–Hcy categories to subsequent mortality [68]. These findings add longitudinal outcome evidence; they do not externally validate a common four-category classifier, establish a causal interaction, or demonstrate phenotype-specific treatment benefit. Definitions, populations, adjustment, and outcomes differ (Supplementary Table S1).

### 8.2. Reference and Discordant Phenotypes

The normal/normal phenotype is a reference category; its incremental prognostic information, like that of the other categories, has not been established. The low vitamin D/normal homocysteine (“vitamin D-dominant”) phenotype may reflect deficiency from low sunlight, seasonality, obesity-related dilution, dietary insufficiency, or malabsorption [31,32,33,34]; these are explanatory possibilities rather than evidence of a vascular risk category. The normal vitamin D/high homocysteine (“homocysteine-dominant”) phenotype motivates assessment of alternative determinants, including folate, vitamin B12, vitamin B6, renal function, thyroid function, smoking, alcohol, medications, and genetic factors [12,13,35,37,38]. These differential considerations derive from established biomarker biology, not from validated clinical utility of the proposed phenotype classification.

### 8.3. The Concordant Low Vitamin D and High Homocysteine Phenotype

The low vitamin D/high homocysteine profile is a candidate subgroup for study. “Concordant adverse” describes the directions of the biomarker abnormalities; observed risk associations in selected cohorts do not establish a transportable clinical risk category [68]. Individuals in this category may have overlapping nutritional insufficiency, renal-metabolic burden, inflammation, oxidative stress, obesity-related vitamin D dilution, impaired one-carbon metabolism, or endothelial dysfunction [23,24,25,26,27,28,29,30,38,47]. Direct prospective validation for cardiovascular outcomes is limited, and the profile should not be interpreted as proof that vitamin D deficiency caused hyperhomocysteinemia or that correcting both biomarkers will prevent events. Its proposed value is to enable explicit comparison of joint and separate biomarker associations. Stroke-oriented research is informed mainly by homocysteine-specific evidence and preliminary vitamin D–Hcy observational findings [67], without establishing a preferred endpoint for phenotype-guided treatment.

### 8.4. Discordance as a Research Question

Discordant profiles allow researchers to test whether each biomarker contributes information independently of the other. Their usefulness must be demonstrated against models containing the individual biomarkers and conventional covariates; descriptive heterogeneity alone does not explain neutral trials. A recurring methodological caution applies throughout: dichotomizing continuous biomarkers at arbitrary thresholds can create artifacts in which apparent group-specific associations reflect the cut point rather than biology. Continuous modeling and restricted cubic splines are therefore preferable, and any dichotomized phenotype should be validated against continuous analyses.

## 9. Requirements Before Clinical Translation

### 9.1. Prospective and Incremental Predictive Validity

Preliminary predictive work should be distinguished from validation of this two-marker classification. A retrospective single-center study of 655 stroke patients included vitamin D and Hcy within a seven-biomarker model for 90-day disability and reported internal discrimination, calibration, and decision-curve performance [69]. It did not isolate the incremental contribution of the proposed vitamin D–Hcy categories or provide external validation; some reported AUC differences were internally inconsistent. Similarly, joint-category mortality associations [68] do not by themselves establish incremental prediction beyond accepted risk models. Future evaluation should compare a conventional model, that model plus each biomarker separately, and a joint model with a prespecified interaction, using incident outcomes and external validation. Clinical reclassification thresholds and uncertainty should be prespecified. Routine paired screening is not supported by the current evidence.

### 9.2. Subclinical Atherosclerosis and Vascular Aging as Candidate Endpoints

Subclinical atherosclerosis and vascular aging may be more suitable early-research endpoints than major events. Coronary artery calcium, carotid plaque and intima-media thickness, arterial stiffness, endothelial function, and microvascular imaging may capture earlier changes related to metabolic and inflammatory pathways [4,14,16,40,41,42,47,48]. Prospective external validation and continuous, confounder-adjusted analyses are required before combined profiles are used for prediction. These measures differ in biological specificity and validation as surrogates. Their use should follow the mechanism being tested, and a change in an intermediate endpoint would not by itself establish clinical benefit.

### 9.3. Clinical Utility and Cost-Effectiveness

Even reproducible incremental prediction would not establish clinical utility. A prospective impact study would need to show that a prespecified action informed by paired testing improves patient-important outcomes compared with established care, while accounting for testing costs, false classification, downstream interventions, and harms. Decision-curve analysis can inform potential net benefit but cannot substitute for such evaluation; cost-effectiveness also remains untested. Until these requirements are met, the proposed categories should guide research questions rather than screening or treatment decisions.

## 10. Causal Roles, Effect Modification, and Methodological Issues

Covariate handling must follow the estimand and temporal ordering. For the total causal effect of baseline 25(OH)D on subsequent Hcy, prior renal dysfunction, B-vitamin status, adiposity, and season may confound the association if they affect both exposure and outcome. If the question instead concerns vitamin D and vascular events, Hcy or inflammation could lie on a proposed mediating pathway; automatic adjustment would then remove part of the total effect. These roles are hypotheses to specify, not intrinsic properties of a variable. Conditioning on a common consequence of exposure and disease, such as selected hospital attendance, or on post-treatment response can introduce collider bias. A confounder-adjusted model and a mediation model therefore answer different questions.

### 10.1. Nutritional and Genetic Determinants

Folate, vitamin B12, and vitamin B6 are central to homocysteine metabolism, so studies that do not measure B-vitamin status are difficult to interpret: a low vitamin D/high homocysteine phenotype may reflect combined nutritional insufficiency rather than a vitamin D–specific effect, and high homocysteine with normal vitamin D may be driven almost entirely by B-vitamin status or renal function [12,13,37,38]. Genetic variation compounds this. MTHFR C677T affects folate-dependent remethylation, particularly in low-folate contexts [35], and CSPPT illustrates how nutrient interventions may depend on baseline metabolic and genetic context [21]. Vitamin D pathway genes (VDR, CYP2R1, CYP27B1, CYP24A1, GC) may influence metabolism and supplementation response; variants in CYP2R1, CYP24A1, and VDR have been reported to modify the efficacy of vitamin D3 supplementation for raising 25(OH)D [36]. Genetic factors are best positioned as research variables rather than immediate clinical tools.

### 10.2. Renal Function

Renal function is a major shared determinant and potential confounder because the kidney participates in vitamin D activation and homocysteine metabolism; CKD also affects mineral metabolism, inflammation, and vascular calcification [1,2,3,12,13]. Baseline eGFR merits measurement and often adjustment when it precedes and influences both biomarkers or the outcome. It should not be included automatically in every causal model: renal changes downstream of an exposure or intervention may be mediators, and the timing and estimand must be specified. CKD subgroup or effect-modification analyses require a prespecified rationale and interaction test; measuring albuminuria, cystatin C, disease stage, or active vitamin D therapy may clarify context without requiring stratification by all of them.

### 10.3. Obesity and Adiposity

Obesity complicates vitamin D interpretation because lower 25(OH)D may reflect volumetric dilution, sequestration, or altered metabolism rather than simple deficiency; BMI alone may be insufficient, and waist circumference, visceral adiposity, and body composition may be more informative [31,32,33,34]. Obesity may also modify supplementation response [34]. Future studies should test whether the association differs by adiposity, insulin resistance, and inflammatory markers.

### 10.4. Lifestyle, Seasonality, and Measurement

Sunlight and outdoor activity, smoking, alcohol, diet quality, sleep, and shift work influence one or both markers; smoking is particularly relevant to higher homocysteine. Seasonality is a major issue, since a single 25(OH)D measurement varies by season, latitude, and behavior. Assay variability further affects 25(OH)D, while homocysteine is sensitive to preanalytic conditions, fasting, sample handling, renal function, and medications, and deficiency/elevation cutoffs vary across studies. Studies should record sampling season, use standardized assays and prespecified cutoffs, apply continuous and categorical definitions in sensitivity analyses, and, where possible, repeat measurements.

### 10.5. Reverse Causality and Residual Confounding

Reverse causality cannot be excluded in cross-sectional studies: poor health reduces outdoor activity and lowers vitamin D; chronic kidney disease and inflammation raise homocysteine; and chronic disease alters diet, supplementation, and metabolism. Even extensive adjustment cannot remove residual confounding. Observational evidence should therefore be used to generate hypotheses rather than to justify treatment claims. Prospective cohorts, Mendelian randomization, mechanistic studies, and phenotype-based trials are needed to clarify whether specific links are causal or prognostic beyond shared determinants.

For prediction, renal function, B vitamins, adiposity, inflammation, and season may be candidate predictors if available at the intended prediction time; selection and shrinkage should serve out-of-sample performance rather than be interpreted as confounder control. Descriptive analyses should report biomarker distributions and age-, sex-, season-, or disease-standardized summaries without causal claims. Effect modification requires a prespecified exposure-by-modifier term and scale-specific estimates with uncertainty; separate significance in one subgroup and not another does not demonstrate interaction.

## 11. Discussion of Competing and Complementary Explanations

The four explanations below are non-exclusive and have unequal support. The shared-determinant explanation has the broadest epidemiological and physiological basis. Forward and reverse regulatory hypotheses have narrower experimental support, and vascular effect modification has limited, outcome-specific observational support requiring replication. Exposure-specific MR and outcome trials constrain interpretation but do not directly adjudicate a unified axis.

### 11.1. Direct Regulation: Preclinical Support and Limited Human Biomarker Evidence

VDR–Nrf2–MTR and NFE2L2–MTHFR regulation is supported in myocardial experimental systems [43,44], with VDR–CBS effects also demonstrated in osteoblast models [46]. Human intervention results are mixed [29,30,62,63,64,65] and do not establish the experimental mechanism as a mediator of response. This model remains plausible, not established as a reciprocal human vascular mechanism.

### 11.2. Reverse Regulation: Preclinical Support

Hcy-related CYP24A1 upregulation and myocardial VDR suppression were demonstrated in rat and cardiomyocyte models [45]. Human longitudinal or interventional confirmation of this reverse pathway is lacking, and circulating 25(OH)D cannot be substituted for the active-vitamin-D and tissue-signaling measures studied.

### 11.3. Shared Determinants: Broader Epidemiological and Physiological Support

Renal dysfunction, nutritional status, adiposity, illness, and health behavior provide established determinants capable of producing co-occurrence [12,13,23,24,25,26,27,28,29,30,31,32,33,34]. This explanation has broader human support than reciprocal regulation, although the proportion of the observed association it explains is unresolved. It does not prove why any particular trial was neutral.

### 11.4. Effect Modification: Outcome-Specific Observations and an Unvalidated Vascular Hypothesis

Interaction was reported for executive function [38] and homocysteine was identified as an effect modifier in an incident-stroke analysis [67], whereas CAD interaction tests were null [28]. A mortality study reported joint-category associations [68], which are not equivalent to replicated biological synergy. These heterogeneous findings require independent replication with prespecified additive and multiplicative tests; differential response to combined nutritional treatment remains unestablished.

### 11.5. What the Integration Establishes and Leaves Unresolved

The contribution of this integration is an evidence map that identifies where apparently connected findings stop short of validating the next link. Experimental regulation, human biomarker co-occurrence, and exposure-specific stroke signals answer different questions. Shared determinants remain an alternative to a unified causal explanation; a reciprocal human system, externally validated incremental prediction by the two-marker classification, and phenotype-specific treatment benefit have not been demonstrated. Conceptual coherence is therefore a basis for discriminating tests, not evidence of biological convergence.

## 12. Future Directions for Testing Combined Biomarker Hypotheses

### 12.1. A Staged and Feasible Research Agenda

Stage 1 is prospective validation using repeated biomarkers and incident outcomes, with external validation and comparison against each marker alone and conventional predictors. Stage 2 is a focused mechanistic or biomarker trial testing one primary hypothesis, with stable cointerventions and measurements matched to the pathway. Only reproducible, clinically relevant signals from these stages would justify considering a large event-driven trial. Stroke or small-vessel disease may be candidates because of homocysteine-specific evidence [49,50,51,52], alongside limited observational paired-marker findings [67], but this does not identify a preferred endpoint for the combined profile. An enriched trial alone would not establish that phenotype-guided selection is better than standard selection; that question requires an appropriate comparison across selection strategies or a prespecified treatment-by-profile interaction.

Table 3 distinguishes established measurement considerations from author-proposed design choices and identifies the statistical requirements for each stage.

### 12.2. Biomarker-Response and Mechanistic Integration

Focused trials should measure achieved 25(OH)D and Hcy to document biological separation, with a prespecified primary biomarker or mechanistic endpoint. A null result could reflect inadequate biological separation, but it may also indicate no clinically meaningful effect—both possibilities should be evaluated. This issue is especially relevant in obesity, where achieved 25(OH)D differs by body size [31,32,33,34], and in chronic kidney disease, where homocysteine may remain elevated despite supplementation. Folate, vitamin B12, vitamin B6, and renal function are central biological variables rather than optional covariates [12,13,37,38]. Cross-design comparisons should seek explanations for discordance as well as agreement. Achieved biomarker values are post-randomization variables. Response-based analyses are exploratory unless appropriately designed, because selecting responders can break the balance created by randomization; the randomized comparison should remain primary.

### 12.3. Genetic, Epigenetic, and Intermediate-Endpoint Priorities

MTHFR genotype, VDR and vitamin D pathway variants, and methylation markers are optional mechanistic variables to test [35,36], although genetic findings should not be translated prematurely into clinical testing. Because homocysteine and one-carbon metabolism are closely linked to methylation capacity, the intersection of vitamin D, homocysteine, methylation, and vascular aging is a hypothesis for focused early research. Early mechanistic studies could select intermediate endpoints such as endothelial function, arterial stiffness, inflammatory markers, or microvascular measures according to the pathway under investigation. Such outcomes are not interchangeable, and biomarker or imaging improvement is not a substitute for demonstrating patient-important benefit. The need for an event trial should depend on the preceding evidence, feasibility, and remaining clinical uncertainty.

### 12.4. Power, Multiplicity, and Progression Criteria

Measuring multiple determinants does not require creating strata for every combination. Simultaneous subdivision by both biomarkers, B-vitamin status, CKD, adiposity, and genotype would produce sparse cells and substantially increase recruitment needs. Each study should specify one primary question, endpoint, and analysis; sample-size calculations should reflect the expected effect, outcome frequency, measurement error, attrition, and any primary interaction, which generally requires greater power than a main effect. Limit secondary interactions, specify the additive or multiplicative scale, and control multiplicity or label analyses exploratory. Prespecified progression criteria should address reproducibility, effect size and precision, feasibility, and safety before an event trial is contemplated. Precision nutrition remains a research aim whose added benefit must be demonstrated, rather than a conclusion drawn from neutral supplementation trials.

## 13. Conclusions

Vitamin D deficiency and hyperhomocysteinemia are each associated with cardiometabolic risk, but both fields have been challenged by neutral or inconsistent randomized trials. The relationship should therefore not be framed as a simple pathway in which low vitamin D raises homocysteine and supplementation prevents cardiovascular disease; that claim exceeds current evidence [6,7,8,9,10,11,12,13,14,15,16,17,18,19,20,21,22,59,61]. Separate preclinical experiments support forward and reverse molecular effects [43,44,45,46], whereas human intervention findings for Hcy are heterogeneous and do not establish vascular benefit [29,30,62,63,64,65,66]. MR provides outcome-specific support for a homocysteine–stroke relationship, with remaining validity and multiplicity concerns, while vitamin D findings are predominantly null but not uniform [49,50,51,52,53,54,55]. CSPPT demonstrates context-specific folic-acid benefit for first-stroke prevention in a low-folate hypertensive population [21], but it does not validate the combined axis, dual supplementation, or the claim that neutral trials failed because of poor targeting.

Current evidence does not directly demonstrate that vitamin D and homocysteine constitute a clinically meaningful unified biological axis, or that people with low 25(OH)D and high Hcy derive differential benefit from combined nutritional intervention. The proposed categories describe coexisting abnormalities. Preliminary longitudinal joint associations and broader biomarker prediction models exist [67,68,69], but their incremental value as a common, externally validated two-marker classifier and their treatment utility remain unproven. Shared determinants have broader human support than reciprocal regulation, and cerebrovascular hypotheses remain supported more strongly by homocysteine-specific evidence than by validation of the combined construct. A staged program of prospective validation, focused mechanistic testing, and conditional consideration of outcome trials is justified as hypothesis testing. Routine paired cardiovascular screening or treatment selection cannot be inferred from this review.

## Figures and Tables

**Figure 1:**
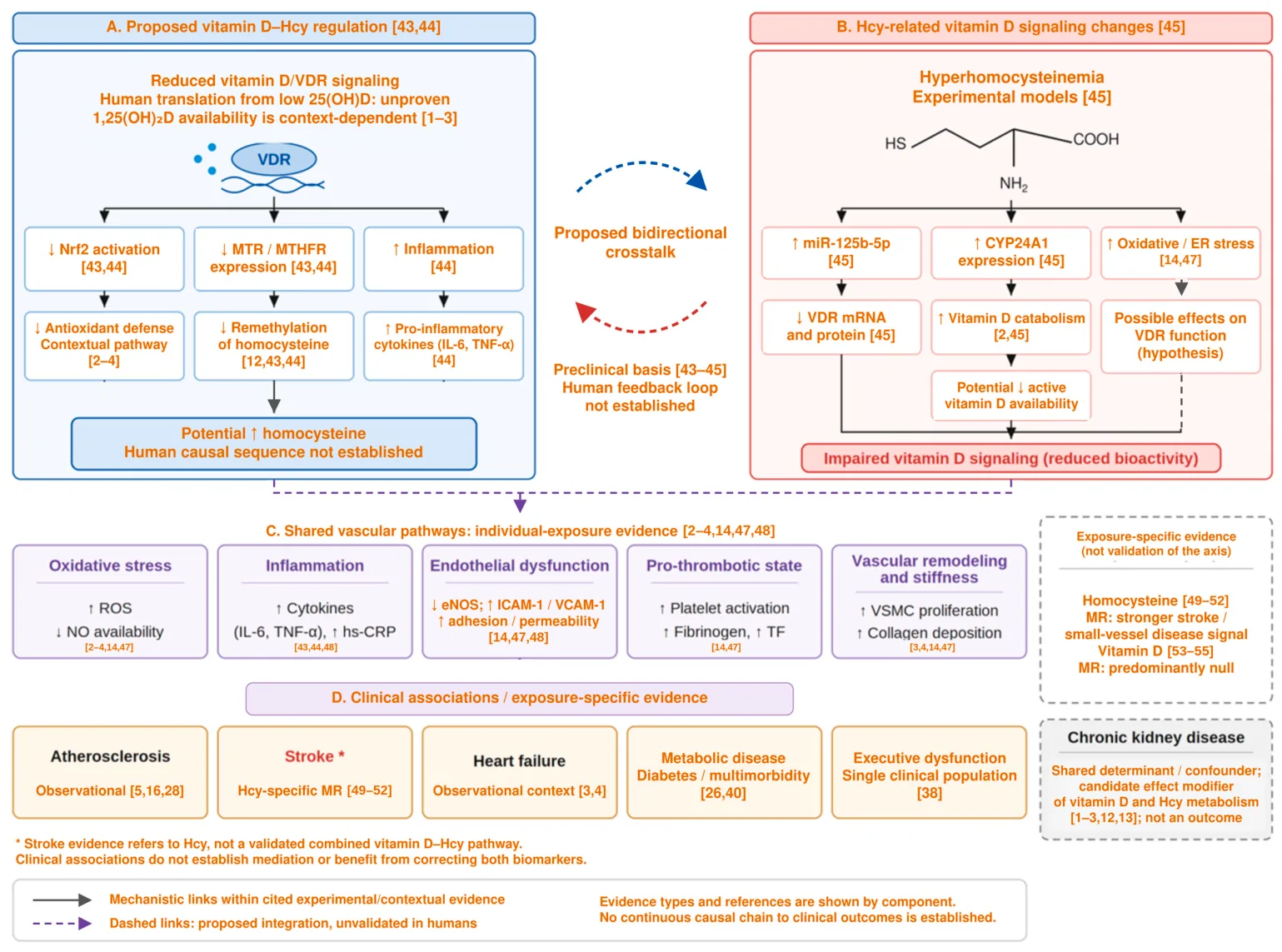
Proposed bidirectional vitamin D–homocysteine crosstalk, shared vascular pathways, and related clinical evidence. This author-developed synthesis is not a validated human causal model. (**A**) The Nrf2–MTR and NFE2L2–MTHFR pathways have preclinical support [43,44]; the depicted low-signaling state and its translation from low circulating 25(OH)D remain hypotheses, and active vitamin D availability is context-dependent [1,2,3]. (**B**) Rat/cardiomyocyte experiments support Hcy-related miR-125b-5p/VDR and CYP24A1 changes [45]. Table A1 details the experimental contexts. Oxidative/ER stress provides mechanistic context [14,47]; its proposed effect on VDR function is shown as hypothetical. (**C**) The vascular pathways are supported by studies of individual exposures, not a demonstrated combined effect [2,3,4,14,43,44,47,48]. (**D**) The retained clinical domains are annotated with observational or exposure-specific genetic evidence [3,4,5,16,26,28,38,40,49,50,51,52], without a causal arrow from panel C. Solid arrows depict mechanistic relationships within the cited experimental or contextual literature; colored dashed links represent unvalidated integration, not proven human mediation. Chronic kidney disease is a shared determinant/confounder and candidate effect modifier [1,2,3,12,13]. * The stronger Hcy–stroke MR signal does not establish the combined axis; vitamin D MR is predominantly null [49,50,51,52,53,54,55]. Abbreviations: 25(OH)D, 25-hydroxyvitamin D; 1,25(OH)_2_D, 1,25-dihydroxyvitamin D; Hcy, homocysteine; VDR, vitamin D receptor; Nrf2/NFE2L2, nuclear factor erythroid 2-related factor 2; MTR, methionine synthase; MTHFR, methylenetetrahydrofolate reductase; CYP24A1, vitamin D 24-hydroxylase; ER, endoplasmic reticulum; ROS, reactive oxygen species; NO, nitric oxide; eNOS, endothelial nitric oxide synthase; ICAM-1/VCAM-1, intercellular/vascular cell adhesion molecule-1; VSMC, vascular smooth muscle cell; TF, tissue factor; IL-6, interleukin-6; TNF-α, tumor necrosis factor-α; hs-CRP, high-sensitivity C-reactive protein; MR, Mendelian randomization.

**Figure 2:**
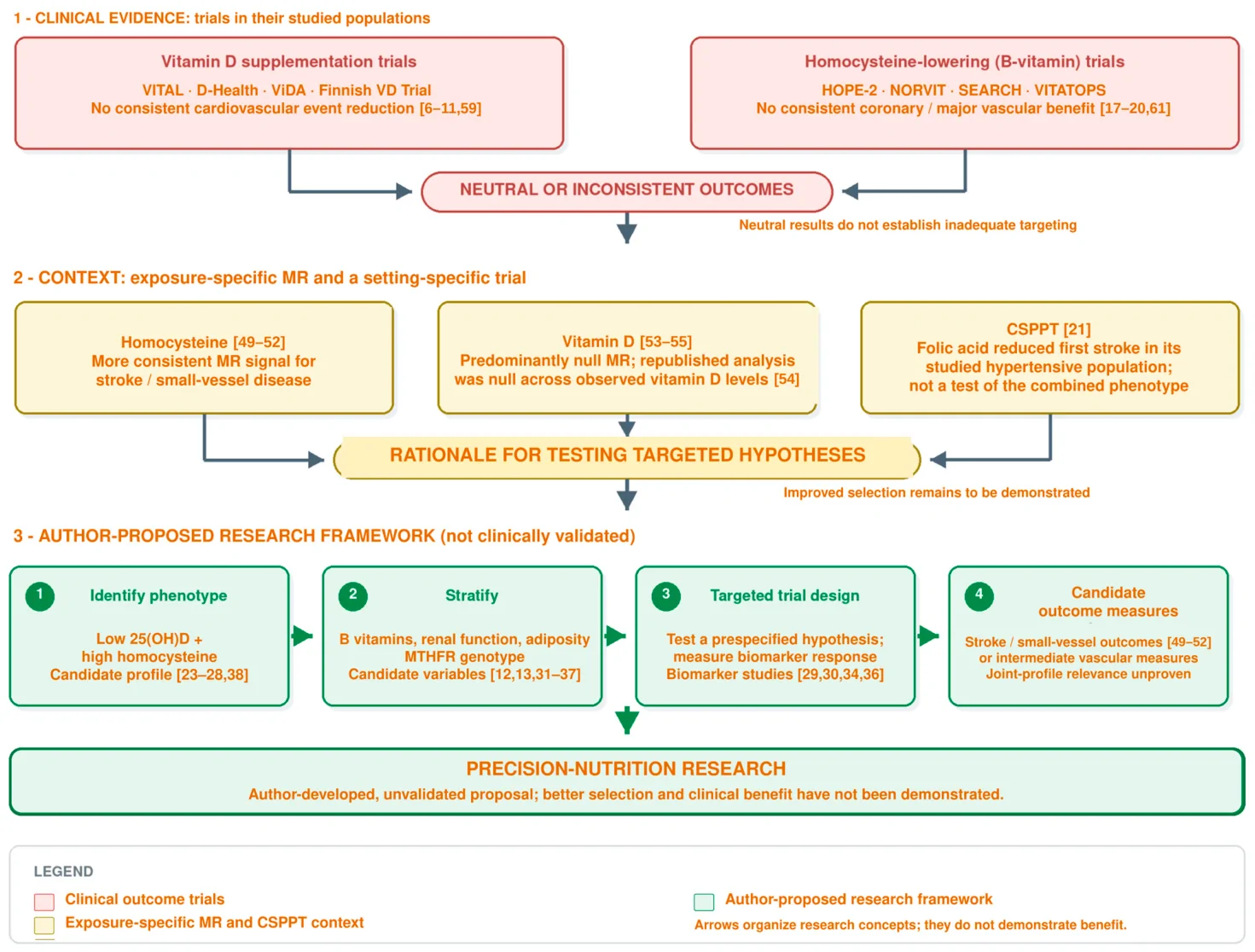
From supplementation evidence to an author-proposed phenotype-based precision-nutrition research framework. The three-stage layout summarizes (**1**) neutral or inconsistent outcomes in the studied vitamin D and B-vitamin trial populations [6,7,8,9,10,11,17,18,19,20,59,61]; (**2**) exposure-specific MR patterns and the setting-specific CSPPT benefit [21,49,50,51,52,53,54,55]; and (**3**) candidate research designs. Vitamin D MR remains predominantly null, including across observed vitamin D levels in the republished analysis [54]; CSPPT did not test the combined phenotype. Candidate profiles are informed by observational biomarker studies [23,24,25,26,27,28,38,67,68], and candidate covariates by nutritional, renal, adiposity, and genetic evidence [12,13,31,32,33,34,35,36,37]. Biomarker-response studies inform measurement choices, not established vascular benefit [29,30,34,36,62,63,64,65,66]; the proposed relevance of stroke or intermediate vascular endpoints to the joint profile remains unvalidated. The framework was developed by the authors and has not been validated as a patient-selection or treatment algorithm. Arrows organize research concepts rather than demonstrate causality, explain neutral trials, or imply improved outcomes. The research sequence proposed in Section 12 is prospective and external validation, followed by focused mechanistic or biomarker trials, and only then consideration of event-driven trials if reproducible evidence warrants them. The three graphic stages summarize evidence domains, not clinical decision steps. Abbreviations: 25(OH)D, 25-hydroxyvitamin D; MTHFR, methylenetetrahydrofolate reductase; MR, Mendelian randomization; CSPPT, China Stroke Primary Prevention Trial.

**Table 2:** Proposed hypothesis-generating classification based on vitamin D and homocysteine status.

Vitamin D Status	Homocysteine Status	Phenotype	Research-Oriented Interpretation	Research Implication
Normal	Normal	Reference phenotype	Reference biomarker category; does not imply low vascular risk or absence of other abnormalities	Comparator phenotype for risk modeling
Low	Normal	Vitamin D-dominant	Consider sunlight, seasonality, obesity, malabsorption, bone/muscle indications	Test whether low 25(OH)D alone adds vascular information
Normal	High	Homocysteine-dominant	Consider folate/B12/B6, renal function, MTHFR, smoking, alcohol, thyroid, medications	Clarify non–vitamin D drivers of hyperhomocysteinemia
Low	High	Concordant low vitamin D/high homocysteine	Coexisting abnormalities may share determinants; greater vascular vulnerability remains a hypothesis	Candidate category for staged prospective validation and focused trials; no routine clinical use

Abbreviations: 25(OH)D, 25-hydroxyvitamin D; B6, vitamin B6; B12, vitamin B12; MTHFR, methylenetetrahydrofolate reductase. No standardized or validated cutoffs exist for this joint cardiovascular classification. “Normal” denotes a prespecified study reference category, not an optimal vascular level. For example, the executive-function study used 25(OH)D < 20 ng/mL and Hcy ≥ 15 μmol/L [38], whereas the angiography study used sample medians of 14.6 ng/mL and 16.2 μmol/L [28]. The mortality analysis instead used 25(OH)D around 50 nmol/L and Hcy around 10 μmol/L to define joint categories [68]. These are study-specific definitions, not recommended thresholds. Future analyses should prespecify cutoffs, assess sensitivity to alternative definitions, and retain continuous modeling. No clinical risk ordering is established.

**Table 3:** Evidence-informed measurements and author-proposed elements of a staged research program.

Element/Status	Evidence or Rationale	Proposed Implementation and Statistical Requirement
Biomarker measurement—evidence-informed	25(OH)D and Hcy reflect nutritional and metabolic determinants [12,13,23,24,25,26,27,28,29,30].	Use standardized assays, repeat measurements, record season and supplement use; prespecify continuous primary analyses and any category cutoffs.
B vitamins, renal function, adiposity—evidence-informed	Established determinants; covariate roles depend on timing and question [12,13,31,32,33,34].	Measure key variables; define adjustment from the estimand (Section 10). Measurement does not mandate simultaneous stratification.
Stage 1: prospective validation—author-proposed	Added prediction by the joint profile remains unproven.	Prespecify incident outcome and conventional comparator model; assess calibration, discrimination and net benefit; use internal and external validation. Plan sample size for events and model complexity.
Stage 2: focused intervention—author-proposed	Biomarker findings are mixed [29,30,62,63,64,65]; pathway mediation is untested.	Specify one primary mechanistic/biomarker endpoint, expected effect, variance, duration, attrition and power; retain intention-to-treat comparison.
Genotype—optional research variable	Variants influence metabolism or biomarker response [35,36]; selection utility is unproven.	Include only for a prespecified hypothesis and sufficient sample size; avoid sparsely populated multivariable strata.
Achieved response—evidence-informed measurement	Documents biological separation; not a validated surrogate or baseline selector.	Measure both biomarkers; avoid conditioning the primary randomized comparison on response. Mediation requires additional assumptions.
Interaction and multiplicity—author-proposed analysis plan	Subgroup significance is not an interaction test; numerous strata reduce power.	Specify the treatment-by-profile or biomarker-by-biomarker term and scale. Power the primary interaction, report CIs, limit secondary tests and control multiplicity.
Stage 3: event trial—conditional author proposal	No combined-profile benefit has been demonstrated; Hcy-specific stroke evidence is indirect.	Proceed only after reproducible prior signals. Prespecify a patient-important primary endpoint; use event-rate and effect-based power calculations and justified follow-up.
Progression/clinical utility—author-proposed	Prediction alone does not establish benefit of testing or treatment.	Set feasibility, precision and safety criteria before progression; assess strategy impact and cost-effectiveness before clinical adoption.

Abbreviations: 25(OH)D, 25-hydroxyvitamin D. These are candidate design elements, not a validated multivariable selection algorithm.

## Data Availability

No new participant-level data were collected or analyzed. The search scope, executed search strategies, aggregate search and selection counts, selection procedures, and source availability and qualitative appraisal are described in Supplementary Materials S1–S4. Additional study summaries are provided in Supplementary Table S1. Published study sources are identified by DOI or PMID; some full texts require publisher access.

## Supplementary Materials

The following supporting information can be downloaded at https://www.mdpi.com/article/10.3390/nu18193167/s1: Supplementary Material S1: Scope and execution; Supplementary Material S2: Complete executed search strategies; Supplementary Material S3: Deduplication and selection; Supplementary Material S4: Source availability and qualitative appraisal; and Supplementary Table S1 describing the additional studies.

## Abbreviations

The following abbreviations are used in this manuscript:

1,25(OH)_2_D	1,25-dihydroxyvitamin D
25(OH)D	25-hydroxyvitamin D
BMI	body mass index
CAC	coronary artery calcium
CI	confidence interval
CKD	chronic kidney disease
CSPPT	China Stroke Primary Prevention Trial
CVD	cardiovascular disease
CYP24A1	cytochrome P450 family 24 subfamily A member 1 (24-hydroxylase)
CYP27B1	cytochrome P450 family 27 subfamily B member 1 (1α-hydroxylase)
CYP2R1	cytochrome P450 family 2 subfamily R member 1 (25-hydroxylase)
eGFR	estimated glomerular filtration rate
GC	group-specific component (vitamin D-binding protein gene)
HOPE-2	Heart Outcomes Prevention Evaluation 2
hs-CRP	high-sensitivity C-reactive protein
IVW	inverse-variance weighted
MR	Mendelian randomization
MTHFR	methylenetetrahydrofolate reductase
MTR	methionine synthase
NFE2L2	nuclear factor erythroid 2-related factor 2 (gene encoding Nrf2)
NORVIT	Norwegian Vitamin Trial
Nrf2	nuclear factor erythroid 2-related factor 2
RCT	randomized controlled trial
RR	relative risk
SEARCH	Study of the Effectiveness of Additional Reductions in Cholesterol and Homocysteine
VDR	vitamin D receptor
VITAL	VITamin D and OmegA-3 TriaL
VITATOPS	VITAmins TO Prevent Stroke

## Appendix A. Selected Study-Level Evidence Underpinning the Proposed Relationship

Table A1, Table A2 and Table A3 retain the original detailed study summaries. Supplementary Table S1 provides the additional experimental, observational, prognostic, and intervention evidence identified in the documented revision search.

These tables summarize the direct mechanistic, human observational, and biomarker-intervention reports cited in the main text. Appraisal is qualitative; no formal risk-of-bias score or certainty grade is assigned. Unverified details are not imputed.

**Table A1:** Principal preclinical studies of directional vitamin D–homocysteine regulation.

**Study/Model**	**Species and Tissue/Cells**	**Perturbation and Pathway**	**Direction and Measured Outcome**	**Directness and Limitations**
Sun et al. 2023 [43]	Mouse myocardium; HL-1 cardiomyocytes; Nrf2-heterozygous mice	Vitamin D/VDR signaling; Nrf2 gain/loss of function; MTR promoter assays	Forward: Nrf2-dependent MTR upregulation and lower Hcy; Nrf2 deficiency attenuated the response.	Experimental pathway support in myocardial systems. No human vascular validation or event endpoint; exact doses/group sizes not extracted from the accessible abstract.
Liu et al. 2024 [44]	Mouse myocardium; HL-1 cells; NFE2L2-heterozygous mice	Vitamin D/active vitamin D during Hcy exposure; NFE2L2 or MTHFR perturbation; promoter assays	Forward: VDR–NFE2L2–MTHFR regulation; reduced Hcy and myocardial inflammatory responses.	Mechanistic intervention supports the pathway within the model. Clinical mediation and cross-tissue generalizability unknown; doses/group sizes not verified from the accessible abstract.
Qi et al. 2025 [45]	Methionine-fed rats; myocardium and renal CYP24A1; cultured cardiomyocytes	Hcy exposure; VDR/miR-125b-5p gain/loss of function; VDR agonist; calcineurin/NFATc4 signaling	Reverse: increased CYP24A1, lower active vitamin D, miR-125b-5p-mediated VDR suppression and cardiac hypertrophy.	Demonstrates selected tissue effects, not a reciprocal human 25(OH)D–Hcy loop. Cultured-cell species, exact doses and group sizes not verified from the accessible abstract.

Abbreviations: VDR, vitamin D receptor; Hcy, homocysteine; Nrf2/NFE2L2, nuclear factor erythroid 2-related factor 2; MTR, methionine synthase; MTHFR, methylenetetrahydrofolate reductase; CYP24A1, vitamin D 24-hydroxylase; NFATc4, nuclear factor of activated T cells 4. Study details are limited to verified primary reports/abstracts; missing extraction fields are not negative quality findings.

**Table A2:** Human observational evidence: design, measurement, effects, and confounder coverage.

**Study/Population/Design**	**Biomarker Assays**	**Representative Estimate**	**Adjustment/Key Determinant Coverage**	**Interpretive Limits**
Amer & Qayyum 2014 [24] 14,630 US NHANES adults; cross-sectional	25(OH)D: DiaSorin RIA; Hcy: Abbott AxSYM fluorescence polarization immunoassay	Per 10 ng/mL higher 25(OH)D: Hcy −0.49 μmol/L (95% CI −0.67 to −0.31) below/equal to 21 ng/mL; no significant association above.	Age, sex/race and other demographics, BMI, smoking, hypertension, glucose, cholesterol, CRP, renal function, folate and B12. Season not in the reported adjustment set.	Most complete simultaneous biochemical adjustment among these biomarker studies; single-time-point association, not temporality or benefit.
Lee et al. 2024 [23] 1375 Korean screening adults; cross-sectional	25(OH)D: Architect i2000 SR; Hcy: Hitachi 747 autoanalyzer	Adjusted β −0.033; *p* < 0.001.	Age/sex, adiposity, BP, lifestyle, comorbidity, metabolic labs, creatinine, hs-CRP, uric acid and TSH. Folate/B12 and season not adjusted; creatinine used rather than eGFR.	Residual nutritional/seasonal confounding; retrospective screening sample.
Pham et al. 2016 [25] 4475 Canadian preventive-program participants; repeated measures; median 1.1 years	25(OH)D: LIAISON chemiluminescence; Hcy: cyclic enzymatic method	Rise in 25(OH)D ≥75 nmol/L vs. no rise: OR 0.32 (0.19–0.54) for Hcy >13 μmol/L at follow-up.	Baseline Hcy, age/sex, BMI, hypertension, LDL, smoking/alcohol, activity/change and B12 at both visits. Renal sensitivity analysis restricted to 4168 with preserved function; folate, season and inflammation not in main model.	Concurrent supplementation and behavior changes; repeated observations do not establish an isolated vitamin D effect.
Mao et al. 2016 [26] 9311 screened; 9254 analyzed: T2DM 839, hypertension 490, CVD 7925; retrospective clinical series	25(OH)D2/D3 and Hcy: LC–MS/MS; folate/B12: immunoassay	Inverse correlations and stratified gradients; no single fully adjusted vitamin D–Hcy effect estimate.	Age/disease and vitamin-stratified analyses; folate/B12 measured. Creatinine >120 μmol/L excluded. No comprehensive eGFR, season or inflammatory adjustment.	Very old clinical population; nutritional and renal restriction is not equivalent to multivariable confounder control.
Kim et al. 2022 [27] 518 Korean screening adults; cross-sectional	25(OH)D: DiaSorin RIA; Hcy: Abbott fluorescence polarization immunoassay	Standardized β −0.106; *p* = 0.022 in the adjusted model relating 25(OH)D to Hcy.	Age, sex, BMI, hypertension and T2DM. Folate/B12, eGFR, season and inflammation not in the reported adjustment set.	Limited covariate control; selection through screening/CT; different modeled outcome from several other studies.
Verdoia et al. 2021 [28] 3150 angiography patients reported; cross-sectional biomarker/angiographic analysis	25(OH)D: LIAISON chemiluminescence; Hcy: laboratory method referenced to prior publication	r = −0.092; low 25(OH)D (first quartile vs. others): OR 1.79 (1.37–2.33) for Hcy above median. Interaction *p* = 0.73 for CAD, 0.78 for severe CAD.	Final Hcy model: age, sex, renal failure, acute coronary syndrome, hemoglobin and vitamin D. Folate/B12 and season not included; CRP measured but not retained in this final model.	Selected disease population; no prospective prediction. Reported quartile strata sum to 3104, below the stated total; denominators are not reconciled here.
Tan et al. 2026 [38] 452 older hypertensive inpatients; cross-sectional	25(OH)D: Beckman Access; Hcy: Diazyme enzymatic cycling; folate/B12 measured	Both abnormalities vs. neither: executive dysfunction OR 2.1 (1.5–2.9); multiplicative interaction *p* = 0.03.	Age, sex, education, BMI, BP, diabetes, eGFR, smoking, supplement use and season; acute illness/infection context in additional models. Measured folate/B12 not listed in the core covariate set.	Neurocognitive outcome, acute-care selection and residual confounding; no incident vascular outcome or external predictive validation.

Abbreviations: RIA, radioimmunoassay; LC–MS/MS, liquid chromatography–tandem mass spectrometry; Hcy, homocysteine; OR, odds ratio; CI, confidence interval; CRP, C-reactive protein; hs-CRP, high-sensitivity C-reactive protein; eGFR, estimated glomerular filtration rate; T2DM, type 2 diabetes mellitus; CAD, coronary artery disease; CVD, cardiovascular disease; BP, blood pressure; BMI, body mass index; LDL, low-density lipoprotein; TSH, thyroid-stimulating hormone. Effect scales and model directions differ. “Not included” refers to the stated model, not necessarily absence of measurement. No study is designated free of residual confounding.

**Table A3:** Linked vitamin D supplementation reports measuring homocysteine.

**Report and Sample**	**Intervention/Baseline Status**	**Biomarker Result**	**Appraisal**
Al-Bayyari 2018 [29] Double-blind placebo-controlled report: 100 overweight reproductive-age women, 50 per group.	Vitamin D3 50,000 IU/week for 2 months. Registration NCT03310307.	Hcy decreased versus placebo (*p* ≤ 0.05); exact baseline and change estimates not available in the verified abstract.	Biomarker study; no cardiovascular events. The later publication reports the same participants.
Al-Bayyari 2021 [30] Post hoc report: 120 randomized; 20 lost; 2 placebo outliers excluded; 98 analyzed (50/48).	Same dose/duration. Baseline 25(OH)D 8.4/8.6 ng/mL and Hcy 18.9/17.9 μmol/L (vitamin D/placebo). Normal folate/B12 required.	At 2 months Hcy 11.9/17.9 μmol/L; unadjusted endpoint difference −6.0 μmol/L (*p* < 0.001).	Attrition/exclusions; post hoc analysis, reporting inconsistencies and narrow population. No independent replication, pathway mediation, or outcome benefit.

Values for the later report are from its treatment-group tables. Its abstract gives a different allocation count from the Methods/flow diagram; the latter is used here. Detailed B-vitamin concentrations and an adjusted Hcy treatment effect with CI were not reported in the extracted tables. Linked reports should not be counted twice. Dose descriptions are research details, not clinical recommendations.
