# Supplementary material for: The Proposed Vitamin D–Homocysteine Relationship in Cardiometabolic Health: A Narrative Review of Mechanisms, Evidence, and Research Phenotypes
Source: Nutrients. 2026 Sep 25;18(19):3167. doi: 10.3390/nu18193167 (PMC13667118; doi:10.3390/nu18193167)
Supplement: Supplementary file 1 [file nutrients-18-03167-s001.zip › nutrients-4572661-supplementary S2.pdf]

S2 Complete executed search strategies

The broad A searches define the retrieved record pool. PubMed B–D are nested subsets used to describe the original conceptual domains; their counts must not be added to A. Expanded Europe PMC pilot syntax was rejected after response/sentinel checks; only the final explicit phrase query below contributes to the audit counts. The pilot amendment is preserved in the audit archive.

PubMed A — 487 records

("Vitamin D"[Mesh] OR "vitamin D"[tiab] OR "25-hydroxyvitamin D"[tiab] OR "25-hydroxyvitamin D3"[tiab] OR "25(OH)D"[tiab] OR calcifediol[tiab] OR calcitriol[tiab] OR cholecalciferol[tiab] OR "vitamin D receptor"[tiab] OR VDR[tiab]) AND ("Homocysteine"[Mesh] OR "Hyperhomocysteinemia"[Mesh] OR homocysteine[tiab] OR hyperhomocysteinemia[tiab] OR hyperhomocysteinaemia[tiab]) AND ("1800/01/01"[Date - Publication] : "2026/07/31"[Date - Publication])

Execution timestamp (UTC): 2026-09-21T12:24:42.678399+00:00. Interface: NCBI ESearch; all identifiers exported and article metadata retrieved with EFetch.

PubMed B — 160 records

("Vitamin D"[Mesh] OR "vitamin D"[tiab] OR "25-hydroxyvitamin D"[tiab] OR "25-hydroxyvitamin D3"[tiab] OR "25(OH)D"[tiab] OR calcifediol[tiab] OR calcitriol[tiab] OR cholecalciferol[tiab] OR "vitamin D receptor"[tiab] OR VDR[tiab]) AND ("Homocysteine"[Mesh] OR "Hyperhomocysteinemia"[Mesh] OR homocysteine[tiab] OR hyperhomocysteinemia[tiab] OR hyperhomocysteinaemia[tiab]) AND ("1800/01/01"[Date - Publication] : "2026/07/31"[Date - Publication]) AND ("cardiovascular"[tiab] OR "cardiometabolic"[tiab] OR "stroke"[tiab] OR "cerebrovascular"[tiab] OR "endothelial"[tiab] OR "atherosclerosis"[tiab])

Execution timestamp (UTC): 2026-09-21T12:25:29.472038+00:00. Interface: NCBI ESearch; all identifiers exported and article metadata retrieved with EFetch.

PubMed C — 273 records

("Vitamin D"[Mesh] OR "vitamin D"[tiab] OR "25-hydroxyvitamin D"[tiab] OR "25-hydroxyvitamin D3"[tiab] OR "25(OH)D"[tiab] OR calcifediol[tiab] OR calcitriol[tiab] OR cholecalciferol[tiab] OR "vitamin D receptor"[tiab] OR VDR[tiab]) AND ("Homocysteine"[Mesh] OR "Hyperhomocysteinemia"[Mesh] OR homocysteine[tiab] OR hyperhomocysteinemia[tiab] OR hyperhomocysteinaemia[tiab]) AND ("1800/01/01"[Date - Publication] : "2026/07/31"[Date - Publication]) AND ("Mendelian randomization"[tiab] OR "Mendelian randomisation"[tiab] OR "randomized"[tiab] OR "randomised"[tiab] OR "trial"[tiab] OR "supplementation"[tiab] OR "folate"[tiab] OR "vitamin B12"[tiab] OR "vitamin B6"[tiab])

Execution timestamp (UTC): 2026-09-21T12:25:44.301063+00:00. Interface: NCBI ESearch; all identifiers exported and article metadata retrieved with EFetch.

PubMed D — 55 records

("Vitamin D"[Mesh] OR "vitamin D"[tiab] OR "25-hydroxyvitamin D"[tiab] OR "25-hydroxyvitamin D3"[tiab] OR "25(OH)D"[tiab] OR calcifediol[tiab] OR calcitriol[tiab] OR cholecalciferol[tiab] OR "vitamin D receptor"[tiab] OR VDR[tiab]) AND ("Homocysteine"[Mesh] OR "Hyperhomocysteinemia"[Mesh] OR homocysteine[tiab] OR hyperhomocysteinemia[tiab] OR hyperhomocysteinaemia[tiab]) AND ("1800/01/01"[Date - Publication] : "2026/07/31"[Date - Publication]) AND ("combined"[tiab] OR "joint"[tiab] OR "interaction"[tiab] OR "phenotype"[tiab] OR "double risk"[tiab] OR "precision nutrition"[tiab])

Execution timestamp (UTC): 2026-09-21T12:25:52.874817+00:00. Interface: NCBI ESearch; all identifiers exported and article metadata retrieved with EFetch.

EuropePMC A — 371 records

TITLE_ABS:"vitamin D" AND TITLE_ABS:homocysteine AND FIRST_PDATE:[1800-01-01 TO 2026-07-31]

Execution timestamp (UTC): 2026-09-21T12:29:32.095084+00:00. Interface: Europe PMC REST search; all 371 records exported in one response.
