# Supplementary material for: The Proposed Vitamin D–Homocysteine Relationship in Cardiometabolic Health: A Narrative Review of Mechanisms, Evidence, and Research Phenotypes
Source: Nutrients. 2026 Sep 25;18(19):3167. doi: 10.3390/nu18193167 (PMC13667118; doi:10.3390/nu18193167)
Supplement: Supplementary file 1 [file nutrients-18-03167-s001.zip › nutrients-4572661-Supplementary S1.pdf]

S1 Scope and execution

This supplement documents the actual revision search and the resulting evidence audit for a narrative review. It does not reconstruct historical searches or convert the article into a systematic review. The initial narrative references concerning individual biomarkers, major cardiovascular trials, and MR remain selected contextual evidence. The supplementary search specifically targeted the relationship between vitamin D and homocysteine, without requiring a vascular term in the broad query.

PubMed was queried through NCBI E-utilities and Europe PMC through its REST API. Both searches were executed on 21 September 2026; publication-date limits retained the manuscript cutoff of 31 July 2026. No unverified historical execution date is assigned. Searches were not restricted by language, age, species, or study design at retrieval; selection prioritized English-language primary reports relevant to adult cardiometabolic interpretation. PubMed uses its publication-date field and Europe PMC its FIRST_PDATE field; indexing and publication-date conventions differ.

Scopus, Web of Science, and Google Scholar were not rerun. Europe PMC substantially overlaps PubMed and provides a complementary phrase search, not independent database coverage. This focused scope may miss studies using other terminology or indexed elsewhere. Search counts are snapshots at execution and may change with indexing.
