# Supplementary material for: The Proposed Vitamin D–Homocysteine Relationship in Cardiometabolic Health: A Narrative Review of Mechanisms, Evidence, and Research Phenotypes
Source: Nutrients. 2026 Sep 25;18(19):3167. doi: 10.3390/nu18193167 (PMC13667118; doi:10.3390/nu18193167)
Supplement: Supplementary file 1 [file nutrients-18-03167-s001.zip › nutrients-4572661-supplementary S3.pdf]

## S3 Deduplication and selection

Records were merged in source order. Exact PMID was the first matching key, followed by normalized DOI and then normalized title plus year. HTML entities and formatting were removed from displayed titles. Duplicate database records were mapped to one audit identifier. Two publications reporting the same randomized trial were kept as distinct publications and linked as one study when interpreting evidence.

| Stage | Number |
|---|---|
| PubMed broad A records | 487 |
| Europe PMC final phrase records | 371 |
| Total database records | 858 |
| Duplicate database records removed | 344 |
| Unique records screened | 514 |
| Not selected for the focused synthesis | 386 |
| Related contextual records not expanded | 105 |
| Selected detailed publications | 23 |
| Already detailed in Tables A1–A3 | 12 |
| Newly added reports [46,56,57,62–69] | 11 |

The 386 not-selected records comprise 59 population/reproductive-focus records, 111 secondary/case-level/protocol/commentary records, 170 topic/endpoint or background-covariate records, 27 preprints/conference abstracts/dissertations, and 19 non-English reports. Reasons are mutually exclusive according to the recorded decision order. The 105 contextual records are not described as ineligible. Their bibliographic entries and selection notes were reviewed by both authors together with the remaining record-level screening decisions.

AI tools were used to assist initial title/abstract screening. Titles were assessed first; abstracts or relevant abstract content were considered where relevance required clarification. Both authors (Y.-A.L. and S.-G.K.) reviewed every preliminary screening decision for all 514 unique records, including selection for detailed synthesis, retention as contextual evidence, and non-selection. The corresponding author (S.-G.K.) additionally verified the publications selected for inclusion in the detailed synthesis. The raw identifiers, metadata, and record-level decisions provide an audit trail for the selections. Final inclusion decisions were made by the authors, who retained responsibility for scientific verification.

Prioritization was based on directness, experimental intervention or temporal outcome data, confounder characterization, sample size/precision, and whether a report materially changed an existing interpretation. Positive and null results were retained. The selected set is representative: it should not be interpreted as all studies meeting systematic eligibility criteria. The 23 selected publications correspond to 22 study groupings because references 29 and 30 report the same trial; other overlapping source populations are not assumed independent.
