# Supplementary material for: The Proposed Vitamin D–Homocysteine Relationship in Cardiometabolic Health: A Narrative Review of Mechanisms, Evidence, and Research Phenotypes
Source: Nutrients. 2026 Sep 25;18(19):3167. doi: 10.3390/nu18193167 (PMC13667118; doi:10.3390/nu18193167)
Supplement: Supplementary file 1 [file nutrients-18-03167-s001.zip › nutrients-4572661-supplementary Table S1.pdf]

Supplementary Table S1. Additional studies identified in the revision search

Study	Design and exposure	Relevant result	Appraisal and source basis
Kriebitzsch et al. 2011 [46] PMID 21898591	Murine MC3T3-E1 and primary VDR-knockout osteoblasts; human SaOS-2 cell line. LASA observational component, n=1264.	Active vitamin D induced CBS expression and transsulfuration; VDR knockout abolished induction. LASA data suggested a non-linear 25(OH)D–Hcy association.	Full text. Direct experimental forward-pathway support, not reciprocal vascular regulation. Human cell line is osteosarcoma-derived. LASA adjusted for age, sex and creatinine; no intervention or vascular outcome.
García-Bailo et al. 2013 [56] PMID 23742939	Canadian Health Measures Survey; n=1928, ages 16–79; cross-sectional.	No significant 25(OH)D–Hcy association after adjustment including sex, waist circumference, activity, hormone use and season.	Full text. Null population result. Age omitted for collinearity; no simultaneous renal/folate/B12 control. Biomarker association, not incident outcomes.
Blondon et al. 2016 [57] PMID 27023449	MESA cross-sectional biomarker analysis; n=6443 for Hcy; multiethnic adults without CVD.	Per 10 ng/mL lower 25(OH)D, Hcy was 3.7% higher (95% CI 3.0–4.3).	Full text. Demographic, adiposity, behavioral and GFR adjustment; folate/B12 intake sensitivity analysis. Estimated intake is not measured B-vitamin status. Cross-sectional association does not establish mediation.
Zhou et al. 2022 [67] PMID 35551015	Nested case-control study: 1079 incident ischemic stroke cases and 1079 matched controls in rural China.	Below 25(OH)D 20 ng/mL, OR per SD increase 0.85 (0.73–0.99); upper vs lower tertile OR 0.61 (0.41–0.89). Homocysteine was reported as an effect modifier.	Primary abstract. Exact interaction estimate, full adjustment set and multiplicity handling not verified from full text. Adds prospective-source outcome evidence, not external validation of the proposed classifier.
Chen et al. 2025 [68] PMID 41473830	NHANES 2001–2006; 5459 hypertensive adults; H-type subgroup n=2276; mortality linkage through 2019.	Joint categories were associated with mortality. Within H-type hypertension, per 10 nmol/L higher vitamin D, adjusted all-cause HR 0.942 (0.906–0.980) and CVD HR 0.927 (0.878–0.978).	Full text. Demographic, lifestyle, comorbidity, folate and B12 adjustment; eGFR/season absent from listed final covariates. Observational mediation and joint categories are not causal proof. No external classifier or treatment validation.
Xu et al. 2026 [69] PMID 42306622	Retrospective single-center acute ischemic stroke cohort, n=655; 90-day disability; seven vitamin-related biomarkers.	Reported internal test-set AUC 0.884 for biomarker panel vs 0.734 for clinical model; calibration and decision-curve analyses included.	Full text. Panel includes more than vitamin D/Hcy; isolated two-marker contribution not established. Internal validation only. Reported delta-AUC values are inconsistent with displayed AUCs; no external or treatment-impact validation.

Study	Design and exposure	Relevant result	Appraisal and source basis
Imanparast et al. 2020 [62] PMID 32971450	Four-arm randomized placebo-controlled trial, n=92 with T2DM; vitamin D3 50,000 IU/week, chromium 500 µg/day, both, or placebo; four months.	Abstract reports Hcy reductions in the vitamin D, chromium and combination arms. No vascular events studied.	Primary abstract. Baseline 25(OH)D/Hcy, B-vitamin data, attrition and precise between-group Hcy effects not verified. Within-arm significance alone does not quantify treatment effect.
Handayani et al. 2026 [63] PMID 42047069	Placebo-controlled epilepsy trial; 40 randomized, 34 completed (17/arm). Vitamin D3 2000 IU/day for 12 weeks.	Baseline 25(OH)D 19.14/22.60 ng/mL; Hcy 3.55/4.17 µmol/L (D/placebo). Hcy change +7.90/+8.15 µmol/L; between-group p=0.290.	Full text. Per-protocol analysis; attrition; B-vitamin supplements stopped and baseline B-vitamin concentrations not measured. Very low baseline Hcy and medication context limit generalization. No vascular event endpoint.
Liu et al. 2024 [66] PMID 39559875	MCI and vitamin D deficiency; 402 randomized to placebo (135), folic acid (134), or folic acid plus vitamin D3 1600 IU/day (133); 24 weeks.	Primary ITT MoCA difference: combination vs placebo 0.456 (95% CI −0.198 to 1.110), p=0.171; vs folic acid 0.038 (−0.600 to 0.676), p=0.907.	Primary abstract. Cognitive endpoint; exact folate dose and numeric baseline/change Hcy not verified. Does not establish vascular benefit or differential response across paired biomarker categories.
Zang et al. 2024 [65] PMID 39554649	120 older hypertensive patients; H-type subgroup 60, allocated 30/30. Vitamin D2 800 IU/day added to folic acid 5 mg/day and antihypertensive care; 12 weeks.	Post-treatment Hcy 11.03±1.03 vs 13.14±2.04 µmol/L; unadjusted endpoint difference −2.11 µmol/L.	Full text. Described as retrospective yet claims random division; allocation and blinding unclear. Statistical/text inconsistencies; baseline arm-specific Hcy not given. No hard vascular outcomes or treatment-by-profile interaction.
Bouazza et al. 2022 [64] PMID 35930297	CKD stage 3, n=306; active vitamin D3 regimens of 2000 IU/day or 60,000 IU/month, assessed at 24 and 36 weeks (monthly regimen included loading).	Vitamin D status improved, but Hcy was not reduced according to the report.	Full text. Active-regimen comparison without untreated placebo; numerous outcomes; reporting inconsistencies and uncertain independence from a related report. No demonstrated vascular-event reduction.

Abbreviations: Hcy, homocysteine; CBS, cystathionine β-synthase; VDR, vitamin D receptor; LASA, Longitudinal Aging Study Amsterdam; MESA, Multi-Ethnic Study of Atherosclerosis; MCI, mild cognitive impairment; MoCA, Montreal Cognitive Assessment; ITT, intention-to-treat; CVD, cardiovascular disease; HR, hazard ratio; OR, odds ratio; AUC, area under the receiver operating characteristic curve. Reference numbers correspond to the revised main manuscript. These studies are not pooled and their effect scales are not interchangeable.
