# Supplementary material for: The Proposed Vitamin D–Homocysteine Relationship in Cardiometabolic Health: A Narrative Review of Mechanisms, Evidence, and Research Phenotypes
Source: Nutrients. 2026 Sep 25;18(19):3167. doi: 10.3390/nu18193167 (PMC13667118; doi:10.3390/nu18193167)
Supplement: Supplementary file 1 [file nutrients-18-03167-s001.zip › nutrients-4572661-supplementary S4.pdf]

S4 Source availability and qualitative appraisal

For the eleven additions, full texts were examined for references 46, 56, 57, 63–65, 68, and 69. References 62, 66, and 67 were assessed from verified primary abstracts; details absent from those sources were not imputed. Qualitative appraisal considered design, sample/selection, confounder control, intervention/cointervention, attrition, reporting consistency, precision, clinical directness, and external validity. These narrative judgments are not formal risk-of-bias scores or GRADE ratings. No effect estimates were pooled.
